# Prelacrimal Recess Approach in Unilateral Maxillary Sinus Lesions: What Is the Impact and Efficacy?

**DOI:** 10.3390/medicina60020222

**Published:** 2024-01-27

**Authors:** Mohamed Abdulla, Osama Refaat, Mohamed Alahmer, Ayman Yehia, Hesham Abdelsalam, Khaled Abdelaal, Mohamed Shams Eldin

**Affiliations:** 1 Department of Otorhinolaryngology, Faculty of Medicine, Al-Azhar University, Cairo 11675, Egypt; 2 EPCRS Excellence Center, Plant Pathology and Biotechnology Lab., Faculty of Agriculture, Kafrelsheikh University, Kafr El-Sheikh 33516, Egypt

**Keywords:** prelacrimal recess approach, endoscopic sinus surgery, Lund–Kennedy Endoscopic Scoring System

## Abstract

*Background and Objectives*: Chronic sinusitis is a commonly encountered diagnosis for otorhinolaryngologists. The profound negative effect of rhinosinusitis on patients’ quality of life is frequently overlooked, and surgical lines of treatment are numerous. The aim of the study was to assess the comparative efficacy of endoscopic middle meatal antrostomy with the endoscopic prelacrimal recess approach, combined with middle meatal antrostomy in the treatment of unilateral chronic maxillary sinus lesion. *Materials and Methods*: Thirty patients with unilateral chronic maxillary sinus lesions enrolled in the study at Alahsa hospital. Patients were divided into two groups: 15 treated through a middle meatal antrostomy and 15 treated via a combined middle meatal antrostomy and prelacrimal recess approach. Demographic and clinical information of the patients, including the medical history, CT scan findings, diagnosis, recurrence, and complications, were gathered and analyzed. Pre- and postoperative clinical findings were graded utilizing the Lund–Kennedy Endoscopic Scoring System. *Results*: The enrolled patients varied in age from 18 to 56, with 60% being male and 40% being female. Antrochoanal polyp, maxillary sinus mucocele, and unilateral allergic fungal sinusitis were among the pathological diagnoses. The follow-up period averaged 14.3 months. Following surgery, two patients in Group II encountered nasal discomfort, which included synechia and epiphora. The success rate for preserving a patient’s disease-free condition was 86.7%. A statistically significant difference in disease-free incidence was observed among the patients in group II. In group I, recurrence was identified in 26.7% of the patients. The postoperative symptoms diminished considerably, and the VAS score was reduced substantially. In Group II patients, however, there was no significant difference in scarring. Clinically significant differences were observed in the mean total Lund–Kennedy Endoscopic scores when compared to their preoperative values. *Conclusions*: Achieving endoscopic access to the sinus’s anterior, lateral, inferior, and inferomedial regions is facilitated by operating via the prelacrimal recess, which is the most advantageous approach. This approach facilitates rapid mucosal healing by maintaining the integrity of the nasolacrimal duct and mucosal covering. The specific pathology, surgical objectives, surgeon expertise, and equipment accessibility influence the choice of endoscopic surgical technique.

## 1. Introduction

Due to the placement of the natural ostium and the difficult ascending mucociliary outflow, the maxillary sinus is the sinus most typically impacted by pathology. The function of the maxillary sinus is to significantly warm and humidify the air. Moreover, the maxillary sinus and its floor are utilized in a variety of regenerative procedures that produce a new volume of bone tissue in order to treat bone atrophy [1]. It is also frequently affected by odontogenic infection neoplastic, traumatic, and allergy disorders. Chronic rhinosinusitis (CRS) represents one of the most prevalent diagnoses seen by an otorhinolaryngologist on a daily basis. Chronic sinusitis refers to repeated episodes of inflammation in the sinus membrane that lines the paranasal canal. This condition lasts for a minimum of 8–12 weeks and is characterized by symptoms such as congested airways, rhinorrhea, facial pain, and a disturbed sense of smell [2]. The significant detrimental impact of rhinosinusitis on patients’ quality of life is often underestimated and neglected. Many people were previously informed that they simply had to “live with” their sinus disease [3]. The socioeconomic effect of rhinosinusitis is an increasing subject of study. Conservative estimates US CRS expenses at over USD 30 billion per year, including USD 20 billion in indirect costs. Direct expenditures include medical visits, prescription medications, and surgery, whereas indirect costs entail lost productivity in rhinosinusitis patients [4]. Current recommendations provide precise therapeutic indications for the treatment of CRS. Regarding surgical indication, sinus surgery should be considered for individuals with CRS who are resistant to medicinal therapy. The choice to operate should be considered in patients with symptomatic illness, excluding those with real or imminent complications, according to the recent guidelines [5]. Since the 1980s, functional endoscopic sinus surgery (FESS) has had several designations to describe its surgical spectrum. The minimally invasive sinus technique, first published in 1996, involves simple sinus ventilation and has been shown to alleviate situations even with severe disease [5]. FESS offers the benefit of maintaining sinus ventilation and mucociliary clearance in cases involving inflammation or infection of the sinuses. Resection of the attachment site should be performed in cases of benign sinonasal tumors, such as inverted papillomas or recurrent antrochoanal polyps, in order to prevent earlier recurrence [6]. In basic situations, a routine uncinectomy and middle meatal antrostomy may be enough for disease visualization and clearance. However, we all too often encounter situations in which we can see substantial maxillary sinus pathology that we do not have a way to eradicate. There are few alternative options in such cases when the usual uncinectomy and middle meatal antrostomy are insufficient [7]. Complete removal of the lesion is frequently challenging due to the instrument’s restricted reach, despite the improved visibility provided by a 70° or 90° endoscope, particularly in cases involving lesions located in the anterior or inferior wall. [8] The removal in such case may be executed utilizing either the conventional Caldwell–Luc approach (CLA) or a canine fossa puncture. In total, 75% of patients have experienced complications associated with the canine fossa approach, which comprise pain, dental complications, and facial numbness [9]. The Caldwell–Luc approach unfortunately, did not provide adequate access to specific maxillary sinus areas and may have resulted in infraorbital nerve injury [10]. Due to the fact that even with specific curved equipment and angled endoscopes or a wide antrostomy diffuse disease in the maxillary sinus, such as recurring polyposis and pathologies originating from the floor or anterior wall of the maxillary sinus, cannot be eliminated fully endoscopically by middle meatal antrostomy [11], Zhou and colleagues introduced the endoscopic intranasal prelacrimal recess approach (PLRA) to the maxillary sinus in 2007. This approach allows for broad access to the walls and recesses of the maxillary sinus while preserving the inferior turbinate and nasolacrimal duct [12]. It affords an uninterrupted view of almost all interior maxillary linings. This enhanced visibility of the operative field may lessen the inherent limitations of endoscopic techniques. PLRA entails surgically generating a passageway by constructing a gap in the medial wall of the prelacrimal recess, which is often one of the most difficult sites to manipulate during conventional middle meatal antrostomy [13]. The aim of this study was to assess the efficacy of endoscopic middle meatal antrostomy versus endoscopic prelacrimal recess approach and middle meatal antrostomy in the treatment of unilateral chronic maxillary sinusitis.

## 2. Materials and Methods

**Figure 1:**
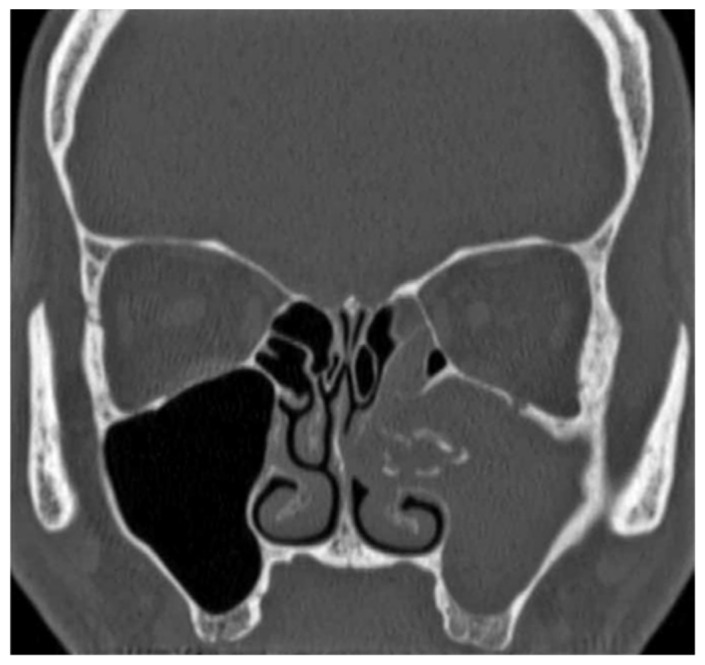
Coronal CT scan of the paranasal sinuses shows the left maxillary and ethmoidal sinuses were completely heterogonous opacified, with linear calcifications and hypertrophy of the maxillary sinus wall; fungal sinusitis.

**Figure 2:**
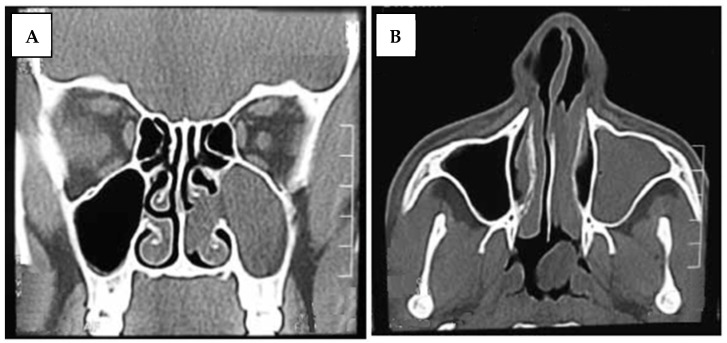
Coronal and axial CT image of an antrochoanal polyp on the left side with extension of the polyp to the nasopharynx (**A**): coronal and (**B**): axial CT image.

**Figure 3:**
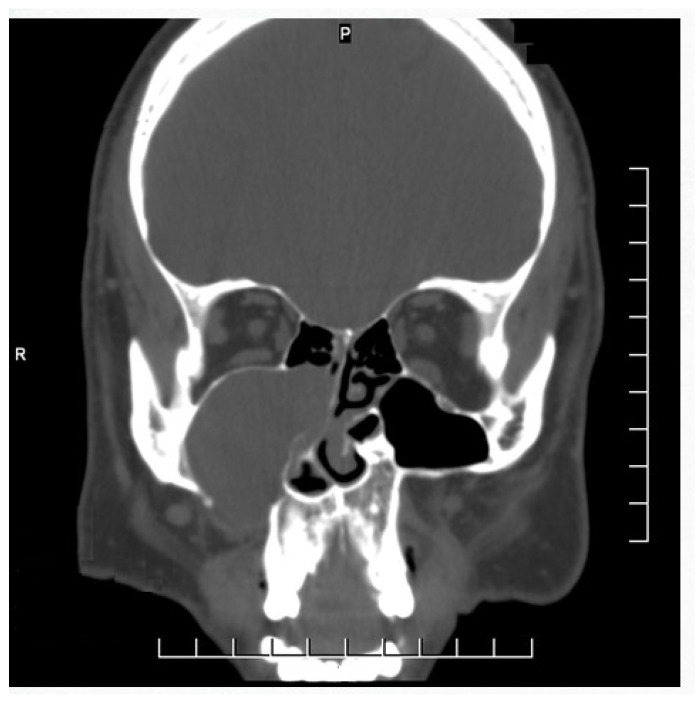
CT scan reveals a right opacified maxillary sinus with medial expansion leading to obstruction of the right nasal cavity and superior bulging causing encroachment on the floor of the orbit.

The study was conducted in the Department of Otorhinolaryngology, (Alahsa hospital) after obtaining the approval of the local ethics committee, and all patients provided informed consent for permission before enrolment in this study. Between November 2019 and December 2022, all selected patients had unilateral chronic maxillary sinusitis and underwent endoscopic sinus surgery. Patients with bilateral sinonasal pathology, sinusitis with complications, allergic rhinosinusitis with nasal polyposis or malignant sinonasal lesions were excluded from the study. Thirty patients were enrolled in the study. They were classified into two groups. Group I included 15 patients treated endoscopically through only a middle meatal antrostomy (MMA), and Group II included 15 patients who were treated via a combined middle meatal antrostomy and prelacrimal recess approach (PLRA). The postoperative care was implemented over a period of 11–23 months, utilizing a nasal endoscope at regular intervals to remove the crust, blood clot, and dry discharge until adequate healing and epithelization were accomplished. Topical nasal corticosteroid and routine saline irrigation were advised for 4 to 6 months after surgery. The demographic profile of the patients as well as their clinical data were obtained including history taking, a local examination, diagnostic nasal endoscopy findings, CT scan findings (Figure 1, Figure 2 and Figure 3), pathological diagnosis, recurrence, and complications.

The 5 main cardinal symptoms were assessed preoperatively, 2 months following the surgery and in the late follow up period ranging from 11 to 23 months including facial pain, headache, nasal obstruction, nasal discharge, and olfactory disturbance. The assessment was conducted using a simple Visual Analogue Scale (VAS), where the patient was asked to give a score of 0 if there were no symptoms and 10 for maximum severity of the symptoms. The score increased from 0 to 10 according to the subjective severity of the symptoms. Preoperative and postoperative findings were also compared in the same sessions regarding scarring degree, crust, mucosal edema, polyps, and nasal discharge depending on the “Lund–Kennedy Endoscopic Scoring System.” These findings were scored from 0 to 2, where a score of 0 was given in the case of no presence, 1 in a mild degree, and 2 in a severe degree of the findings. Both groups’ data were compared at the end of the follow-up period (ranging from 11 to 23 months), and the results were statistically analyzed.

### Surgical Technique

**Figure 4:**
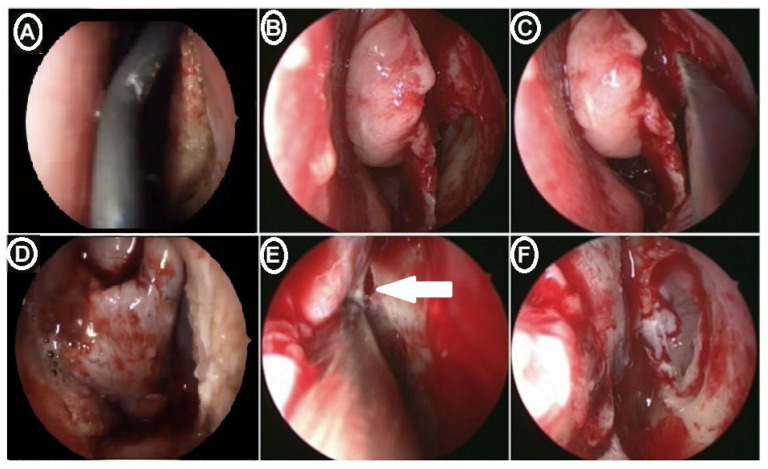
Surgical steps of the prelacrimal recess approach. A c-shaped mucosal incision on the lateral wall of the nasal cavity around the anterior attachment of the inferior turbinate concha (**A**); the mucosa was elevated posteriorly to the insertion site of the inferior turbinate concha (**B**); an osteotomy was performed at the inferior turbinate concha insertion site (**C**); the mucosal flap of the inferior turbinate was sutured to the nasal septum (**D**); the opening of the nasolacrimal duct (labeled with an arrow) was exposed, and the mucosa was left intact (**E**); the maxillary sinus was entered through the antrostomy made at the prelacrimal recess (**F**).

Surgery was conducted under general anesthesia in all instances. Nasal endoscopic surgery started after initial preparation of the nasal cavity by nasal packing with cotton pledges containing Xylometazoline as a topical vasoconstrictor; then, the lateral wall of the nose was infiltrated with 1% lidocaine with 1:100,000 adrenaline solution. It was applied to the lateral nasal wall including the head of the inferior turbinate, inferior meatus, the nasal aperture, the uncinate process, and face of the middle turbinate. A standard uncinectomy with middle meatal antrostomy was performed for patients of both groups with removal of all inflammatory pathological lesions from the maxillary sinus and osteomeatal unit using a 30- and 0-degree endoscope. The prelacrimal recess approach started with a curved mucosal incision (Figure 4A) on the lateral wall of the nasal cavity between the anterior head of the inferior turbinate and the nasal surface of the pyriform aperture and deepened down to the bone, extending from the anterior border of the nasolacrimal dust into the inferior meatus. A mucoperiosteal flap was created and elevated between the inferior meatal wall and lateral nasal wall up to the bony attachment of the inferior turbinate (Figure 4B). At this step, the Hasner’s valve could be identified and preserved. A bony cut was made vertically with an osteotomy passing through the lateral nasal wall starting at the bony insertion site of the inferior turbinate (Figure 4C); then, the cut passed posterosuperiorly to the nasolacrimal duct (NLD) with disconnection of the bony attachment of the inferior turbinate. Once the mucoperiosteum was elevated posteriorly, the bony orifice of the NLD could be considered as a landmark from which the medial part of the prelacrimal recess (PLR), which constitutes the anterior part of the medial wall of the maxillary sinus, was chiseled off. After chiseling the bone posteriorly, the performed flap was medialized exposing the maxillary sinus mucosal wall (Figure 4D). At this this step, the (PLR) was entered by removing the anterior or anteromedial maxillary wall (Figure 4E,F), according to the extension of the present pathology that could be dealt with endoscopically with a wide view that ensured complete and successful interaction. The mucosal flap was then repositioned and closed with vicryl 4 /0 with packing of the nasal cavity using Merocel^®^ nasal pack.

The Statistical Package for the Social Sciences, version 25.0 (IBM SPSS, Armonk, NY, USA: IBM Corp., New York, NY, USA) was used throughout the data analysis process. When it was acceptable to do so, descriptive statistics were given using counts, proportions (percentages), and the mean ± the standard deviation. The chi-square test was used to make a comparison between the middle meatal antrostomy and the middle meatal antrostomy coupled with prelacrimal recess methods among the baseline features of the patients. When calculating statistical significance, a *p* value cutoff point of 0.05 was utilized at 95% confidence intervals. The tests used were the X mean, SD standard deviation: to measure the central tendency of data and the distribution of data around the mean; Student’s t-test: for testing the statistically significant difference between means of two samples; X2 test (chi-square test) to test the statistical relation between different variable or grades (qualitative data). Methods of Statistical Analysis: (i) arithmetic mean: (X) $X=\frac{\Sigma x}{n}$, where Σ = sum, x = value of observation, and *n* = number of observations; (ii) standard deviation = SD, $SD=\sqrt{\frac{\Sigma (X-X{)}^{2}}{n-1}}$, where X = observation, X = mean of the group, and n = number; (iii) Student’s “*t*” test: $t=\frac{X_{1}-X_{2}}{\sqrt{S{D_{1}}^{2}/n_{1}+S{D_{2}}^{2}/n_{2}}}$, where X1 = mean of first group, X2 = mean of the second group, SD1 = standard deviation of the first group, SD2 = standard deviation of the second group, n1= no. of first group, and n2 = no. of second group.

## 3. Results

**Figure 5:**
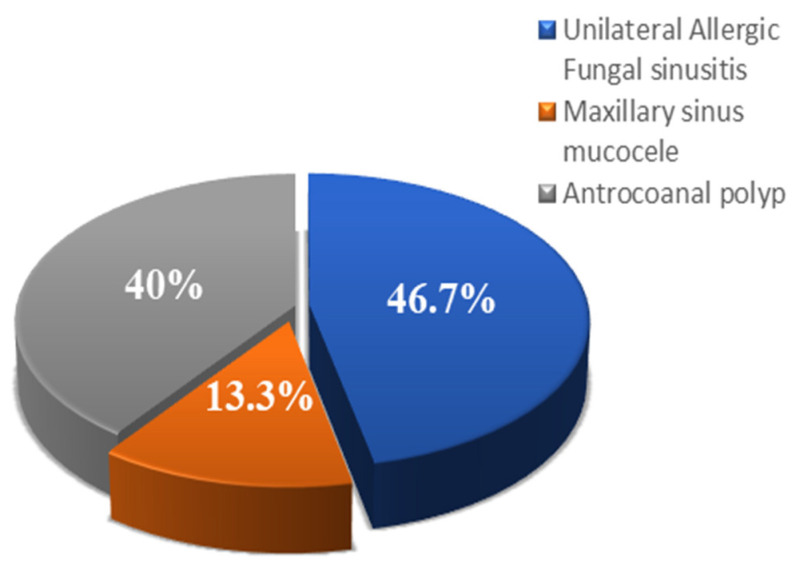
Distribution of the studied cases in relation to their pathological diagnosis.

We conducted research on thirty individuals who had endoscopic sinus surgery after being diagnosed with unilateral chronic maxillary sinusitis and were impacted by the disease. Patients with unilateral chronic maxillary opacity who underwent endoscopic sinus surgery were classified according to the performed endoscopic procedure into Group I, where 15 patients underwent endoscopic sinus surgery using the middle meatal antrostomy approach, Group II, where 15 patients were managed endoscopically through combined approaches (middle meatal antrostomy combined with prelacrimal recess). Following the splitting of the patients participating in this research into two groups, an analysis of their data was carried out. The ages ranged from 18 to 56 years old, with the mean of 39.5 ± 11.5 years old. The most prevalent range of age for people to have surgery for either of these groups was between the ages of 20 and 35. Males made up 60% of the total population, while females only made up 40%. The pathological diagnosis was distributed as follows: unilateral allergic fungal sinusitis (46.7%), maxillary sinus mucocele (40%), and antrochoanal polyp (13.3%) (Figure 5). The mean duration of follow-up following surgeries was 14.3 months (range 11–23 months). In Group II, the inferior turbinate was unstable in two patients after surgery (13.3%), and these patients had nasal discomfort, which improved in the late follow-up. One patient had chronic epiphora, while one other patient had anterior nasal synechia (6.6%). The operation time expanded by 37 min on average when group II was operated on. The thirty patients’ initial demographics and clinical data are presented in (Table 1 and Table 2).

The postoperative period was uneventful for patients in both groups. Overall, an 86.7% success rate was shown in the studied cases of both groups in terms of maintaining a patient’s disease-free status following the procedure. Four patients out of fifteen (26.7%) in group I had recurrence after surgery, but none of the patients in group II showed evidence of recurrence. When compared to Group I, Group II exhibited a statistically significant difference in the incidence of individuals who were disease-free (*p* = 0.032). In group I, the maxillary sinus walls could not be seen to their full extent in eight patients (53.3%), whereas in group II, only two cases (13.3%) presented difficulty with seeing the maxillary sinus in its entirety. In most cases of Group II, the combined prelacrimal and middle meatal antrostomy techniques allowed for complete visualization of the maxillary sinus pathology. Postoperative symptoms were decreased in both approaches, and a significant reduction in the VAS score was obtained in both groups. The improvement in the mean of the symptoms of facial pain, nasal obstruction, nasal discharge, and olfactory disturbance following the combined approach showed a significant statistical difference when compared to that obtained following the endoscopic middle meatal antrostomy approach only. When compared to their preoperative values, the postoperative mean total VAS scores of patients in group I demonstrated a statistically significant and clinically meaningful difference (*p* < 0.0001). According to Table 2, a similar result was seen in patients from group II as well (*p* < 0.0001); however, the postoperative improvement shown in the group II patients was more statistically significant than that seen in the group I patients (*p* = 0.0072), as shown in Table 3 and Table 4. These results indicate a considerable increase in the quality of life after endoscopic surgery, with higher outcomes in favor of patients who utilized a combined approach.

The Lund–Kennedy Endoscopic Scoring System was used to grade the endoscopic findings in the postoperative period. Postoperative changes in nasal discharge and nasal polyps were significantly reduced in both techniques, with a highly statistically significant reduction as compared to the preoperative data (*p* < 0.0001).

Crust and mucosal edema were similarly decreased in the surgical follow-up period of the two groups with a significant statistical difference, although the scarring in the lateral nasal wall in Group II patients revealed no significant statistical difference. The postoperative mean total Lund–Kennedy Endoscopic scores of patients in Group I exhibited a highly statistically significant and clinically relevant difference when compared to their preoperative values (*p* < 0.0001), as shown in Table 5. A similar finding was obtained in group II patients; however, the postoperative improvement shown in the group II patients was statistically significantly greater than that seen in the group I patients (Table 6) (*p* = 0.0323).

## 4. Discussion

Sinonasal disorders could be treated surgically by endoscopic sinus surgery through traditional middle meatal antrostomy as the most basic utilized technique. However, this may not be enough alone, even with an experienced sinus surgeon, as it cannot easily address the entirety of the walls of the maxillary sinus [14]. By combining the prelacrimal approach, which offers enhanced visualization of the maxillary sinus cavity and walls, with middle meatal antrostomy, it is possible to achieve complete eradication of the sinus pathology without any residual pathology. This procedure has the benefit of direct access to the anterior wall of the maxillary sinus without the need to relocate or remove the nasolacrimal duct. Moreover, the inferior turbinate may be restored to its former anatomical position at the completion of the procedure, therefore minimizing the functional complications linked to the excision of the lateral nasal wall. The present research conducted a comparative study of the middle meatal antrostomy and the combination middle meatal antrostomy with prelacrimal recess approach for treating patients with unilateral chronic maxillary sinusitis.

The combination of endoscopic middle meatal antrostomy (EMMA) and prelacrimal recess approach (EMMA-PRA) is more useful than EMMA alone in the treatment of chronic maxillary sinusitis. EMMA had a moderate degree of diagnostic accuracy in patients with acute or chronic sinusitis, according to a study by Kim et al. However, the combined approach of EMMA-PRA produced superior results [15]. It is considered a good option for reaching the infratemporal fossa and lateral part of the pterygopalatine fossa, as well as the anterior maxillary wall and alveolar recess [7]. However, it may not be suitable for direct visualization of the medial part of the pterygopalatine fossa [10]. Following Zhou et al.’s 2007 introduction of PLRA as an innovative technique for managing specific lesions of the maxillary sinus while preserving the NLD and inferior turbinate, the procedure’s efficacy and decreased morbidity have been described and discussed in detail [16]. The prelacrimal approach to maxillary sinus lesions is an excellent method, according to the findings of another study, particularly for pathologies that manifest in the inferior and anterior walls of the maxillary sinus [17].

In the examined cases of both groups, the overall success rate in preserving a patient’s disease-free status after the procedure was 86.7%. Recurrence was observed in four out of fifteen patients (26.7%) in group I following surgery, whereas in group II, no patients exhibited any indications of recurrence. In contrast to Group I, Group II demonstrated a statistically significant disparity in the prevalence of disease-free individuals. (*p* = 0.032) Group I comprised eight patients (53.3%) who exhibited incomplete visibility of the maxillary sinus walls. Conversely, group II comprised only two cases (13.3%) whose surgeons encountered challenges in observing the maxillary sinus in its totality. These findings are close to that obtained in another study where the role of the prelacrimal recess approach in the complete removal of anterior maxillary sinus lesions was evaluated. They conclude that, on using a 0° rigid endoscope, it was possible to observe and control every area. Due to the preservation of the lateral nasal wall, nasal physiological functions, including preserving humidity, warmth, and cleanliness is possible. Their preliminary clinical study revealed that in the concealed regions of the maxillary sinus, remnants would be overlooked in 45% of cases in the absence of the PLRA. Consequently, it is an optimal and minimally invasive method for addressing the challenges associated with the maxillary sinus. [11] Consistent with our research, Line et al. [18] compared endoscopic sinus surgery performed through the prelacrimal recess approach in conjunction with middle meatal antrostomy to conventional middle meatal antrostomy. They found that the prelacrimal recess approach to endoscopic sinus operations did not result in any postoperative complications, including but not limited to facial or cheek puffiness or pyriform aperture stenosis. In some instances, granulations formed at the site of the incision at the head of the inferior turbinate; however, these lesions resolved rapidly [18].

In the current study, postoperative symptoms showed a significant reduction in VAS in both groups of the current study. When compared to the endoscopic middle meatal antrostomy technique alone, the improvement in the mean of the symptoms of face discomfort, nasal blockage, nasal discharge, and olfactory pain after the combination treatment revealed a significant statistical difference. The postoperative mean total VAS scores of patients in Group I revealed a statistically significant and clinically relevant difference when compared to their preoperative values. A similar finding was seen in group II patients; however, the postoperative improvement found in group II patients was statistically significantly higher than that seen in group I patients. The findings demonstrate a significant improvement in the quality of life after endoscopic surgery, particularly for individuals who used a combination strategy. Recent research found that both the middle meatal antrostomy and prelacrimal recess approach considerably decreased postoperative symptoms. However, there was no statistically significant difference between the two procedures in terms of symptom reduction. Complete visualization of maxillary sinus pathology was not possible in 14 (46%) patients who underwent middle meatal antrostomy for maxillary sinus lesions. Conversely, this limitation was observed in six (20%) patients who were treated with the endoscopic prelacrimal approach. Most of the time, the prelacrimal approach successfully achieved the comprehensive visualization of the maxillary sinus pathology. Follow-up results revealed epiphora and numbness in three patients (10%) of the prelacrimal approach group. Postoperative facial pain was reported by five patients (16.6%), while facial edema and swelling were observed in three patients (10%) [17].

The complications encountered in the current study during the mean follow-up time of 14.3 months (range 11–23 months) were seen in group II patients, where the inferior turbinate was unstable in two patients after surgery (13.3%), and these patients had nasal discomfort, which improved in the late follow-up. One patient had chronic epiphora, while one other patient had anterior nasal synechia (6.6%). Acute bleeding, local infections, cosmetic alterations to the nasal ala in the event of substantial bone excision, and disruption to the lacrimal drainage routes with epiphora are all possible consequences of PLRA [19]. Pain or paranesthesia in the ipsilateral sinus and upper incisors accounts for the majority of long-term nasal complications associated with PLRA, with reports ranging from 15.7% to 52.4% of instances [20].

One patient in another series had severe intraoperative bleeding from the maxillary artery, necessitating an immediate switch to a conventional medial maxillectomy for management. Also, seven patients (25%) reported numbness, which improved progressively over the course of four months but did not completely resolve. There were no correlations that were deemed statistically significant between the incidence of postoperative neural morbidity and the osteotomy instrument utilized, the type of pathology treated, the site of origin of the lesion, or the incision of the pyriform aperture. The frequency of early postoperative complications during a 15-day period included cheek swelling in 10% of cases, temporary epiphora, orbital cellulitis, and mild epistaxis in 4% of cases each. All these issues were cured within a month. In contrast, a long-term consequence of this condition was the paresthesia ipsilateral anterior superior alveolar process and teeth. This was recorded in 25% of cases. The symptom exhibited a progressive improvement over the course of a few months in all patients, but it did not completely resolve [21]. The prelacrimal recess technique is an effective method for addressing diseases in the anterior or inferior maxillary wall that are difficult to see using just the middle meatal antrostomy approach. Nevertheless, this method is linked to certain postoperative complications such as postoperative hemorrhage, swelling of the cheeks, numbness of the infraorbital nerve, and epiphora [15].

In the current study, the postoperative endoscopic nasal findings graded by the Lund–Kennedy Endoscopic Scoring System present a highly statistically significant reduction in both groups compared to the preoperative data including nasal discharge, nasal polyps, crust, and mucosal edema. However, patients treated with the combined techniques showed no significant statistical difference in relation to the scarring of the lateral nasal wall. A significant and statistically relevant difference was observed in the postoperative mean total Lund–Kennedy Endoscopic scores of patients in Group I, as compared to their preoperative values. A comparable result was observed in patients of group II; nevertheless, the postoperative recovery observed in this group was more statistically significant than that observed in the patients in group I. It is worth mentioning that the published research does not specifically outline the particular application of the Lund–Kennedy Endoscopic Scoring System with the prelacrimal technique. Further studies will be needed to investigate the potential use of that scoring system in the evaluation of the outcome of the prelacrimal approach. A recent study found a statistically significant difference in postoperative crustations between two groups treated with standard endoscopic sinus surgery (ESS) and the prelacrimal approach (PLA) when comparing their 1-, 3-, and 6-month postoperative follow-up periods [22].

The prelacrimal approach to maxillary sinus lesions is an excellent method, according to the findings of another study, particularly for pathologies that manifest in the inferior and anterior walls of the maxillary sinus. The patient’s CT findings, particularly the distance between the anterior maxillary wall and the anterior wall of the lacrimal duct, primarily dictate the approach to the sinus [17].

A number of limitations apply to the present study, including the small sample size and a shortage of prior research on the subject. Furthermore, individual variation is a factor in the subjective character of the variable measurements (VAS score) utilized to compare outcomes. This indicates that a substantial prospective multicenter study is required to supplement our findings.

## 5. Conclusions

The endoscopic prelacrimal recess approach offers notable advantages in comparison to alternative techniques utilized in maxillary sinus surgery. This is primarily due to its ability to facilitate direct endoscopic entry into the maxillary sinus’s anterior, lateral, inferior, and inferomedial regions, thereby granting substantial access to claimed areas. This method also maintains the integrity of the nasolacrimal duct and the mucosal covering, resulting in prompt healing of the mucosa. Furthermore, in situations where the extent of the pneumatization of the maxillary sinus is uncertain, thereby affecting the difficulty of accessing the anterior wall of the sinus via alternative methods, the prelacrimal recess approach may be employed. In general, the utilization of the prelacrimal recess approach together with endoscopic MMA enhances both the accessibility and the results of anterior maxillary sinus surgery. The selection of the endoscopic surgical technique for the maxillary sinus is based on variables such as the particular pathology, surgical objectives, the surgeon’s proficiency and expertise, and the availability of the appropriate equipment.

## Figures and Tables

**Table 1:** Clinicopathological features of the study population (*n* = 30).

Study Variables	*N* (%)
Study group	
Group I	15 (50.0%)
Group II	15 (50.0%)
Age in years (mean ± SD)	39.5 ± 11.5
Sex	
Male	18 (60.0%)
Female	12 (40.0%)
Pathology	
Unilateral allergic fungal sinusitis	14 (46.7%)
Antrochoanal polyp	12 (40.0%)
Maxillary sinus mucocele	04 (13.3%)
Complication	
Yes	0
No	30 (100%)
Outcome	
Disease free	26 (86.7%)
Recurrence	04 (13.3%)

**Table 2:** Comparison between Group I and Group II of the baseline characteristics of the patients (*n* = 30).

Factor	Study Group		*p* Value
	Group I	Group II	
	*N* (%)	*N* (%)	0.715
	(*n* = 15)	(*n* = 15)	
Age in years			
<40 years	07 (46.7%)	08 (53.3%)	1.000
≥40 years	08 (53.3%)	07 (46.7%)	
Sex			
Male	09 (60.0%)	09 (60.0%)	1.000
Female	06 (40.0%)	06 (40.0%)	
Pathology			
Unilateral allergic fungal sinusitis	07 (46.7%)	07 (46.7%)	
Maxillary sinus mucocele	02 (13.3%)	02 (13.3%)	0.032 **
Antrochoanal polyp	06 (40.0%)	06 (40.0%)	
Outcome			0.715
Disease free	11 (73.3%)	15 (100%)	
Recurrence	04 (26.7%)	0	

** Significant at the *p* < 0.05 level.

**Table 3:** Visual analogue score means of the compared groups.

Visual Analogue Score		Facial Pain	Headache	Nasal Obstruction	Nasal Discharge	Olfactory Disturbance	Total VAS
Group (I)	Preoperative	5.1 ± 1.9	4.68 ± 1.51	6.85 ± 1.67	5.61 ± 1.51	3.78 ± 1.33	27.13 ± 5.79
	Postoperative	3.68 ± 1.56	2.09 ± 0.91	2.10 ± 1.29	2.11 ± 1.54	1.8 ± 1.06	12.47 ± 3.68
*p* Value		0.0348 *	<0.0001 **	<0.0001 **	<0.0001 **	0.0001 **	<0.0001 **
Group (II)	Preoperative	5.46 ± 1.76	5.73 ± 1.27	7.73 ± 1.38	5.73 ± 1.83	5.4 ± 1.88	30.06 ± 3.30
	Postoperative	1.73 ± 1.27	1.26 ± 0.96	1.27 ± 1.03	1.87 ± 1.06	2.13 ± 1.35	8.67 ± 3.5
*p* Value		<0.000 **	<0.0001 **	<0.0001 **	<0.0001 **	<0.0001 **	<0.0001 **

* Significant difference, ** highly Significant at the *p* < 0.05 level.

**Table 4:** Comparison between the postoperative VAS means of the studied groups.

	Mean	SD	*t*-Test	*p* Value	SE	95% CI	
						Lower	Upper
GROUP (I)	12.47	±3.68	−2.898	*p* = 0.0072 **	1.311	1.1139	6.4861
GROUP (II)	8.67	±3.5					

** Significant at the *p* < 0.05 level.

**Table 5:** Lund–Kennedy Endoscopic Scoring System of the compared groups.

Lund-Kennedy Endoscopic Scoring System		Scarring	Crust	Mucosal Edema	Polyps	Nasal Discharge	Total Score
Group (I)	Preoperative	0.47 ± 0.64	0.33 ± 0.49	1.53 ± 0.52	1.6 ± 0.83	1.73 ± 0.46	5.66 ± 1.39
	Postoperative	0.93 ± 0.59	0.47 ± 0.52	0.53 ± 0.55	0.4 ± 0.51	0.6 ± 0.51	2.93 ± 0.96
*p* Value		=0.0502 *	=0.4543 *	<0.0001 **	0.0001 **	<0.0001 **	<0.0001 **
Group (II)	Preoperative	0.33 ± 0.49	0.47 ± 0.52	1.73 ± 0.46	1.8 ± 0.41	1.87 ± 0.35	6.2 ± 0.86
	Postoperative	0.53 ± 0.52	0.93 ± 0.6	1.33 ± 0.49	0.27 ± 0.46	0.8 ± 0.41	3.87 ± 1.3
*p* Value		0.2876	=0.0329 *	=0.0288 *	<0.0001^**^	<0.0001 **	<0.0001 **

* Significant difference, ** Significant at the *p* < 0.05 level.

**Table 6:** Comparison between the postoperative means of the Lund–Kennedy Endoscopic Scoring System in the studied groups.

	Mean	SD	*t*-Test	*p* Value	SE	95% CI	
						Lower	Upper
Group (I)	2.93	±0.96				0.0853	1.7947
Group (II)	3.87	±1.3	−2.253	0.0323 *	0.417		

* Significant difference, at the *p* < 0.05 level.

## Data Availability

The data presented in this study are available on request from the corresponding author. The data are not publicly available due to terms of privacy.
